# Vagus nerve stimulation enhances the cholinergic anti-inflammatory pathway to reduce lung injury in acute respiratory distress syndrome via STAT3

**DOI:** 10.1038/s41420-021-00431-1

**Published:** 2021-03-29

**Authors:** Sheng Li, Di Qi, Jia-ni Li, Xin-yu Deng, Dao-xin Wang

**Affiliations:** 1 Department of Respiratory and Critical Care Medicine, The Second Affiliated Hospital of Chongqing Medical University, 400010 Chongqing, China; 2 Department of Neurology, The Second Affiliated Hospital of Chongqing Medical University, 400010 Chongqing, China

**Keywords:** Acute inflammation, Sepsis

## Abstract

The cholinergic anti-inflammatory pathway (CAIP) is important for antagonizing inflammation and treating several diseases, including acute respiratory distress syndrome (ARDS), and is related to vagus nerve integrity. However, its underlying pathophysiological mechanism is still unclear. We hypothesized that CAIP regulates lung injury repair after ARDS through the STAT3 signaling pathway, which is an important downstream effector of α7nAchR. We enhanced CAIP activity by subjecting rats to vagus nerve stimulation (VNS), and administered the α-7 acetylcholine receptor (α7nAchR) agonist and antagonist to determine whether VNS can reduce lung injury by regulating the pulmonary inflammatory response through CAIP. After being subjected to VNS, the secretion of TNF-α and IL-1β was decreased, while the level of IL-10 was increased in the rat model of ARDS. Moreover, VNS treatment reduced lung mRNA levels of M1 macrophage markers, while increased those of M2 macrophage markers. The expression of Caspase-1 decreased, while that of STAT3 increased in lung tissue after VNS treatment. The aforementioned effects of VNS were reversed by cutting the cervical vagus efferent branch and blocking α7nAchR. These findings suggest that VNS inhibits the ARDS inflammatory response by promoting CAIP activity. Next, we used lentivirus knockdown of STAT3 expression to explore the mechanism of VNS through CAIP on lung inflammation in ARDS model rats. VNS activates α7nAchR, increases STAT3 expression, reduces Caspase-1 expression, suppresses inflammation by inhibiting inflammatory pyroptosis and M1 to M2 macrophage transformation, which may constitute the main mechanism of VNS action in ARDS.

## Introduction

Acute respiratory distress syndrome (ARDS), one of the clinically common diseases with high mortality, is characterized by an acute, diffuse, and inflammatory lung injury^1,2^. The pathogenesis of ARDS is complex, and inflammation-mediated injury plays a key role in this process. Hence, regulating the inflammatory response to promote recovery from ARDS has always attracted significant attention from clinicians; however, so far, no intervention measures have had a clear effect^2,3^. An increasing number of studies have found that the cholinergic anti-inflammatory pathway (CAIP) plays an important role in the regulation of ARDS-associated lung injury^4,5^. During the inflammatory response induced by various stimuli, inflammatory mediators trigger CAIP activity and participate in the recovery from ARDS^6^; however, the pathophysiological mechanism is still not clear. The mode of action of CAIP involves the transmission of inflammatory stimulation signals from the afferent branches of the vagus nerve to the nucleus solitum of the brain. This results in the excitation of the dorsal nucleus of the vagus nerve, and then the impulse passes through the efferent branches of the vagus nerve to release acetylcholine^7^. Consequently, acetylcholine binds to the N-shaped acetylcholine receptor, α7nAchR, on the surface of inflammatory or immune cells^8^, thereby playing an antagonistic role against inflammation. α7nAchR is a key node of CAIP, and the administration of α7nAchR agonist could weaken lipopolysaccharide (LPS)-induced ARDS lung injury^9,10^. Vagus nerve stimulation (VNS) is a relatively mature method for the activation of CAIP that involves the electrical stimulation of the efferent branches of the vagus nerve in the neck, thereby promoting the release of acetylcholine from its terminals, which then acts on α7nAchR^10,11^. It has been clinically used in the treatment of illnesses, such as refractory epilepsy, and depression^11,12^.

It has been found that VNS can enhance CAIP activity and promote the recovery from ARDS^13^; however, the underlying mechanism is still not clear. One possible mechanism is that VNS promotes the vagus nerve endings to release acetylcholine that activates the α7nAchR infiltrating inflammatory cells, leading to the downstream activation of STAT3 (refs. ^9,14,15^). This triggers signals that inhibit the secretion of pro-inflammatory cytokines (TNF-α, IL-1β, etc.) and promote the release of anti-inflammatory cytokines (IL-10) by inflammatory cells^14,16^, thereby reducing lung inflammation and injury caused by ARDS^17,18^.

We used an LPS-induced ARDS rat model to observe the changes in macrophage phenotype and expression of inflammatory factors so as to elucidate whether VNS can reduce the inflammatory response in ARDS and promote recovery from lung injury. We also studied the effects of VNS on CAIP, STAT3, and pyroptosis to explore the mechanism through which VNS promotes lung injury repair in order to provide theoretical support for its use in the prevention and treatment of ARDS in a clinical setting.

## Results

### VNS can reduce the inflammatory response in ARDS rat model

**Fig. 1:**
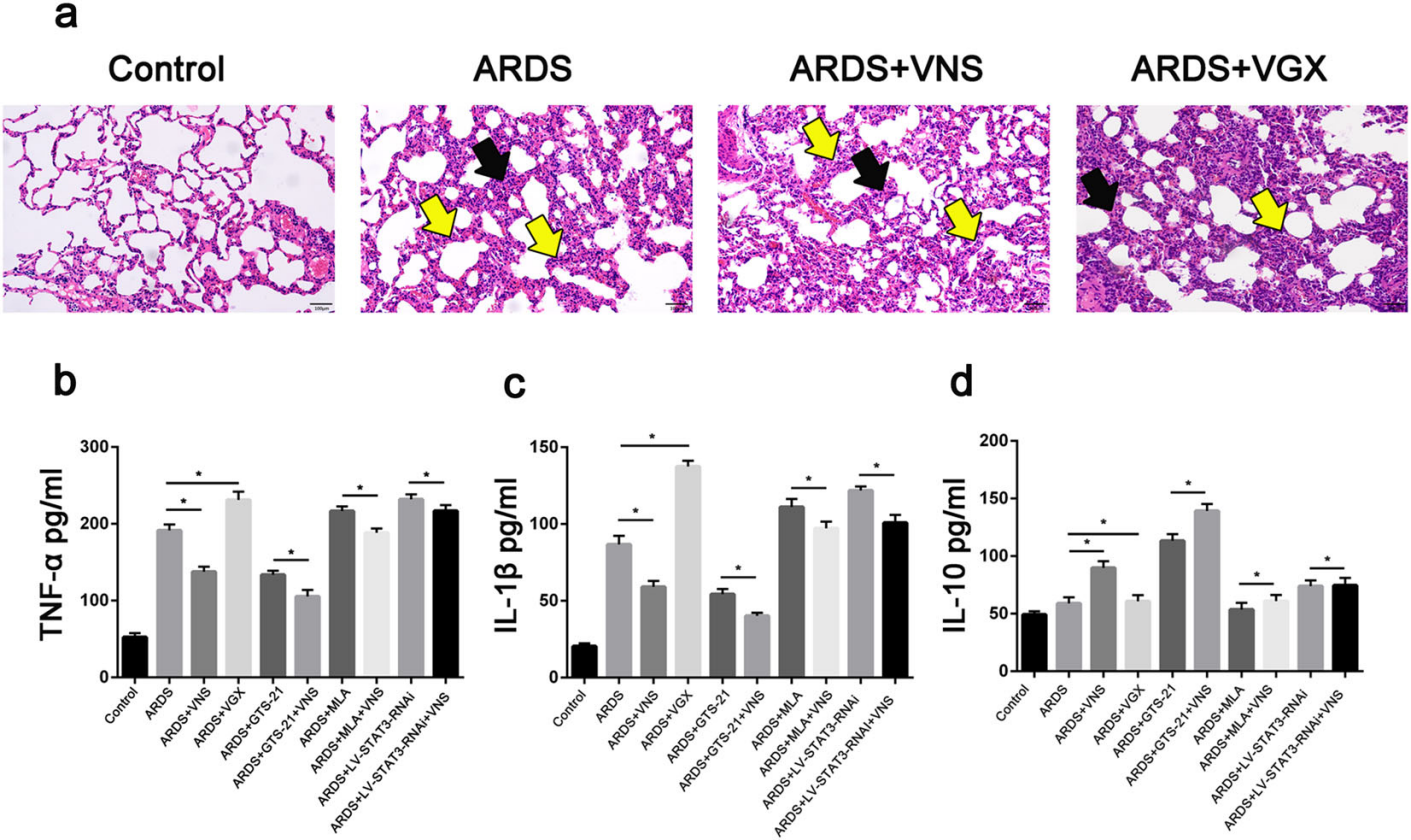
Vagus nerve stimulation (VNS) alleviates inflammation in acute respiratory distress syndrome (ARDS). **a** Hematoxylin and eosin (H&E) staining analysis demonstrated that VNS significantly attenuated LPS-induced lung histopathological alterations (scale bar = 100 μm), while vagotomized (VGX) rats showed increased lung histopathological changes upon LPS stimulation. In the ARDS groups, alveolar walls are moderately thickened (black arrow), alveolar cavities are narrow, and there is increased inflammatory cell infiltration (yellow arrow). In the VNS groups, alveolar walls were slightly thickened (black arrow) and alveolar cavities were narrowed, with a small amount of inflammatory cell infiltration (yellow arrow). In the VGX groups, alveolar walls were severely thickened (black arrow), alveolar cavities were narrow, and there was increased inflammatory cell infiltration (yellow arrow) (*n* = 6 biological replicates). **b**–**d** Effect of acute respiratory distress syndrome (ARDS), vagus nerve stimulation (VNS), vagotomy (VGX), GTS-21, methyllycaconitine citrate (MLA), and LV-STAT3-RNAi treatments on TNF-α (**b**), IL-1β (**c**), and IL-10 (**d**) levels in the bronchoalveolar lavage fluid (BALF). The results are expressed as the mean ± SEM (*n* = 6 biological replicates); *p < 0.05, **p < 0.01.

We administered methyllycaconitine citrate (MLA) or GTS-21 30 min before LPS tracheal instillation, and performed VNS for 10 min after LPS administration for 6 h, and the rats were sacrificed 2 h later. Hematoxylin and eosin (H&E) staining showed a significant alleviation of histopathologic damage in lungs after VNS treatment (Fig. 1a). Enzyme-linked immunosorbent assay (ELISA) results showed a decrease in the levels of the pro-inflammatory cytokines, TNF-α and IL-1β, and an increase in the level of the anti-inflammatory cytokine, IL-10, in the bronchoalveolar lavage fluid (BALF) (Fig. 1b–d); after cutting off the vagus nerve in the neck, the reducing effect of VNS on the inflammatory response was weakened (Fig. 1a–d). GTS-21, an agonist of α7nAchR, can reduce the inflammatory response of ARDS when used alone. After administering GTS-21 to ARDS model rats before VNS, the ELISA results showed a more significant decrease in IL-1β and TNF-α levels, while a significant increase in IL-10 levels was observed (Fig. 1b–d). MLA is an inhibitor of α7nAchR. We subjected the ARDS model rats to MLA treatment alone and found an increase in IL-1β and TNF-α levels, and a decrease in IL-10 levels. When MLA was administered before VNS treatment, the regulatory effects of inflammatory factor expression by VNS were observed to be weakened (Fig. 1b–d). These findings suggest that VNS attenuates the inflammatory response in LPS-induced ARDS model rats by acting on α7nAchR.

### VNS treatment promotes the transformation of macrophages from M1 to M2

**Fig. 2:**
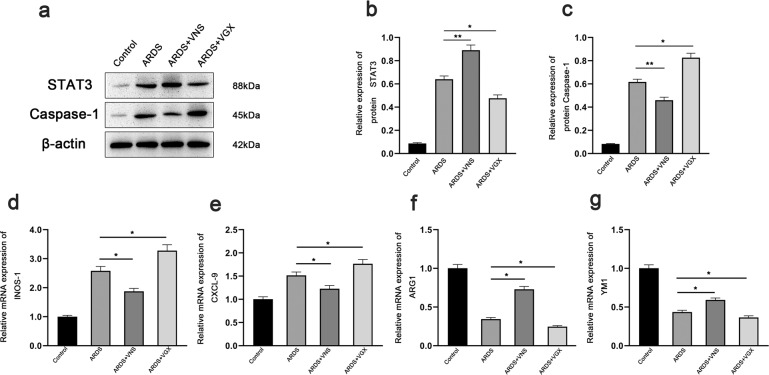
Effects of VNS on STAT3, Caspase-1, and M1M2 macrophage phenotype. **a** Representative western blotting images of STAT3 and Caspase-1 expression in ARDS group, ARDS+VNS group, and ARDS+VGX group (*n* = 6 biological replicates). **b**, **c** Comparison of mean intensity ratios for STAT3 and Caspase-1 in the ARDS group, ARDS+VNS group, and ARDS+VGX group (*n* = 6 biological replicates) through western blotting analysis. **d**–**g** The mRNA levels of the M1 macrophage markers, iNOS and CXCL-9, and those of the M2 macrophage markers, Arg1 and Ym1, were measured in the ARDS group, ARDS+VNS group, and ARDS+VGX group by qPCR and normalized to GAPDH mRNA levels (*n* = 6 biological replicates). The results are expressed as the mean ± SEM (*n* = 6 biological replicates); *p < 0.05,**p < 0.01.

**Fig. 3:**
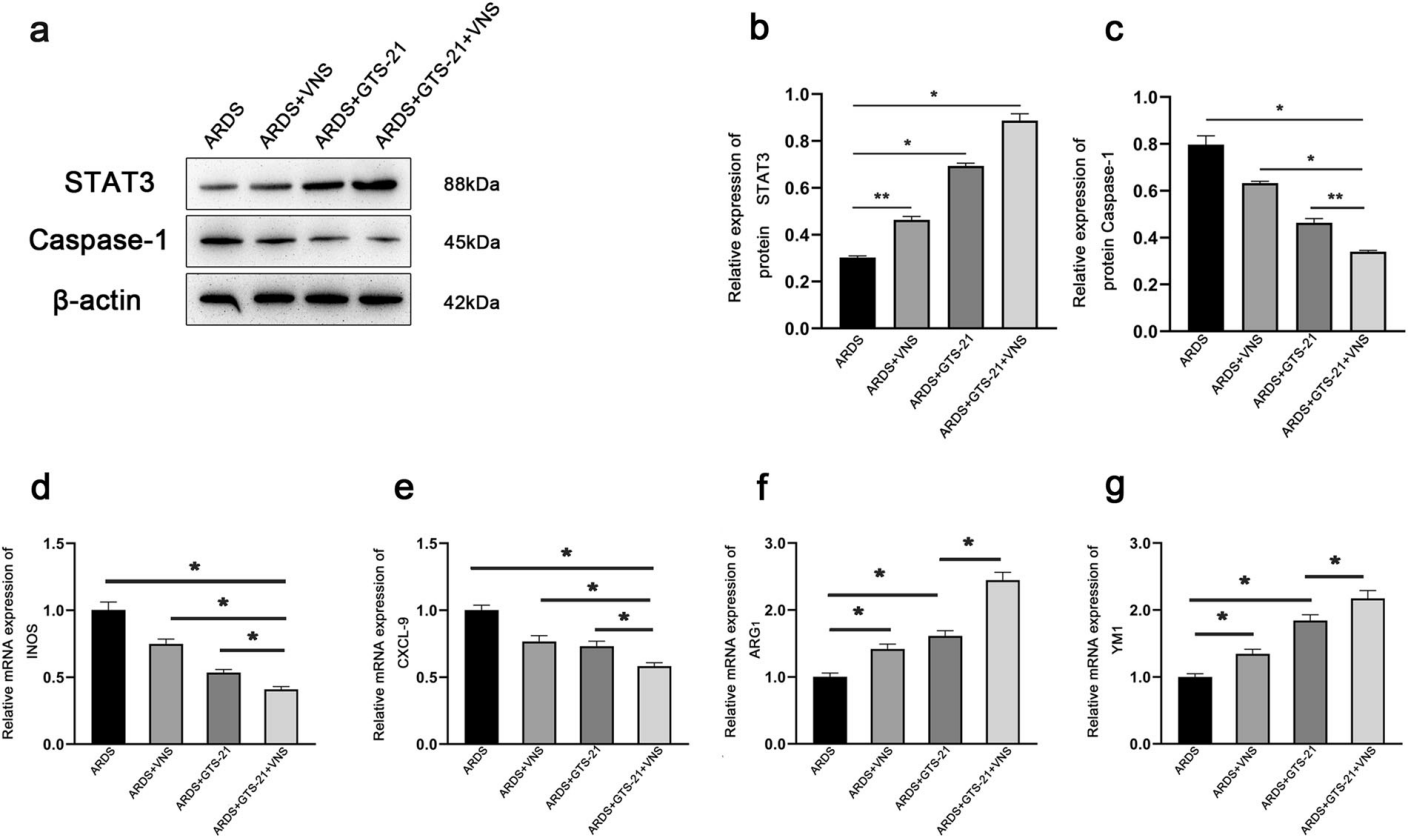
The α7nAchR agonist, GTS-21, can enhance the effects of VNS on STAT3, Caspase-1, and M1M2 macrophage phenotypes. **a** Representative western blotting images of STAT3 and Caspase-1 expression in the ARDS group, ARDS+VNS group, ARDS+GTS-21 group, and ARDS+GTS-21+VNS group (*n* = 6 biological replicates). **b**, **c** Comparison of mean intensity ratios for STAT3 and Caspase-1 in the ARDS group, ARDS+VNS group, ARDS+GTS-21 group, and ARDS+GTS-21+VNS group (*n* = 6 biological replicates). **d**–**g** The mRNA levels of M1 macrophage markers, iNOS and CXCL-9, and the mRNA levels of the M2 macrophage markers, Arg1 and Ym1, were measured in the ARDS group, ARDS+VNS group, ARDS+GTS-21 group, and ARDS+GTS-21+VNS group by qPCR and normalized to GAPDH mRNA levels (*n* = 6 biological replicates). The results are expressed as the mean ± SEM (*n* = 6 biological replicates); *p < 0.05, **p < 0.01.

**Fig. 4:**
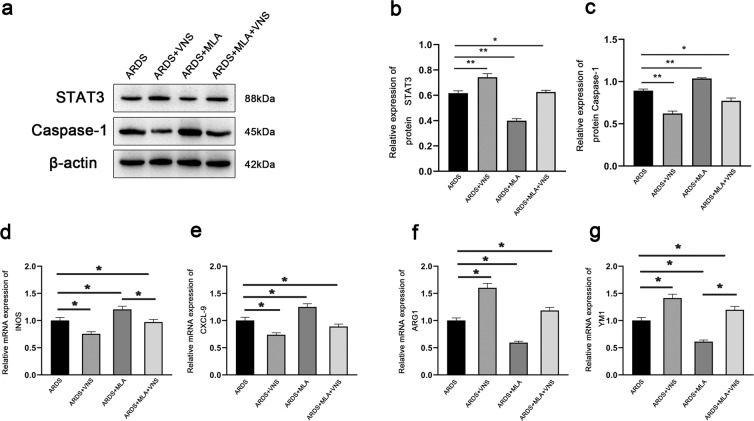
The α7nAchR inhibitor methyllycaconitine citrate (MLA) weakens the effects of VNS on STAT3, Caspase-1, and M1M2 macrophage phenotypes. **a** Representative western blotting images of STAT3 and Caspase-1 expression in the ARDS group, ARDS+VNS group, ARDS+MLA group, and ARDS+MLA+VNS group (*n* = 6 biological replicates). **b**, **c** Comparison of mean intensity ratios for STAT3 and Caspase-1 between the ARDS group, ARDS+VNS group, ARDS+MLA group, and ARDS+MLA+VNS group (*n* = 6 biological replicates). **d**–**g** The mRNA levels of the M1 macrophage markers, iNOS and CXCL-9, and the mRNA levels of M2 macrophage markers, Arg1 and Ym1, were measured in the ARDS group, ARDS+VNS group, ARDS+MLA group, and ARDS+MLA+VNS group by qPCR and normalized to GAPDH mRNA levels (*n* = 6 biological replicates). The results are expressed as the mean ± SEM (*n* = 6 biological replicates); *p < 0.05, **p < 0.01.

In ARDS, lung macrophages with different phenotypes play different roles. Classically activated M1 macrophages express specific markers (CXCL-9, iNOS, etc.), and secrete pro-inflammatory mediators (such as TNF-α)^19^, while alternatively activated M2 macrophages express specific markers (such as YMS-1 and ARG) and secrete anti-inflammatory mediators (such as IL-10)^20,21^. In order to observe the changes in macrophage phenotypes, we evaluated the mRNA levels of CXCL-9, iNOS, YM1, and ARG-1 in the lung tissues of ARDS model rats. After VNS treatment, quantitative real-time PCR analysis showed that the mRNA levels of CXCL-9 and iNOS markers expressed in M1 macrophages was decreased (Fig. 2d, e), while the mRNA levels of ARG-1 and YM1 markers expressed in M2 macrophages was increased (Fig. 2f, g). The α7nAchR agonist, GTS- 21, enhances this transformation (Fig. 3d–g), while the α7nAchR inhibitor MLA attenuates it (Fig. 4d–g), as does cutting off the vagus nerve (Fig. 2d–g). These changes were consistent with the changes in IL-1β, TNF-α, and IL-10 levels observed through ELISA (Fig. 1b–d), suggesting that VNS can promote the transformation of macrophages from M1 to M2 in ARDS rats by acting on α7nAchR, and affect the secretion of inflammatory factors and inflammatory response.

### VNS weakens the pyroptosis in the lung tissue of ARDS rats model by inhibiting the Caspase-1 expression

Pyroptosis is a newly discovered form of programmed cell death with inflammatory effects that involve the inflammatory corpuscles-mediated activation of various Caspases, including Caspase-1, leading to cell perforation and cell death^22–25^. Activated Caspase-1 cleaves the precursors of IL-1β and IL-18 to form active IL-1β and IL-18, which are released outside the cell to disseminate and coordinate inflammatory cell aggregation and amplify the inflammatory response. It has been found that inhibiting the expression of Caspase-1 can weaken the pyroptosis of alveolar endothelial cells^22^. We found that the expression of Caspase-1 in the lung of ARDS model rats was significantly increased, while the expression of Caspase-1 was inhibited after VNS treatment (Fig. 2a, c). This inhibitory effect was enhanced by the administration of the α7nAchR agonist, GTS-21 (Fig. 3a, c), and decreased by the administration of α7nAchR inhibitor, MLA (Fig. 4a, c); the inhibitory effect was also found to be significantly reduced after the vagus nerve was severed (Fig. 2a, c). These changes were consistent with the changes in IL-1β levels observed through ELISA (Fig. 1c). This suggests that VNS can weaken pyroptosis in the lungs by acting on α7nAchR, which may be important for its influence on the inflammatory response.

### VNS plays protective effect on ARDS by STAT3

We used lentivirus to knock down STAT3. We detected STAT3 expression in the lung by western blotting, and a small amount of STAT3 expression was observed in the normal lung tissue. The expression of STAT3 in the lungs of ARDS model rats was increased, which was significantly enhanced after VNS treatment (Fig. 2a, b). The increase in STAT3 expression was more obvious after VNS treatment with α7nAchR agonist, GTS-21 (Fig. 3a, b), while administration of the α7nAchR inhibitor, MLA, and transection of the cervical vagus nerve reduced STAT3 expression (Figs. 2a, b and 4a, b).

**Fig. 5:**
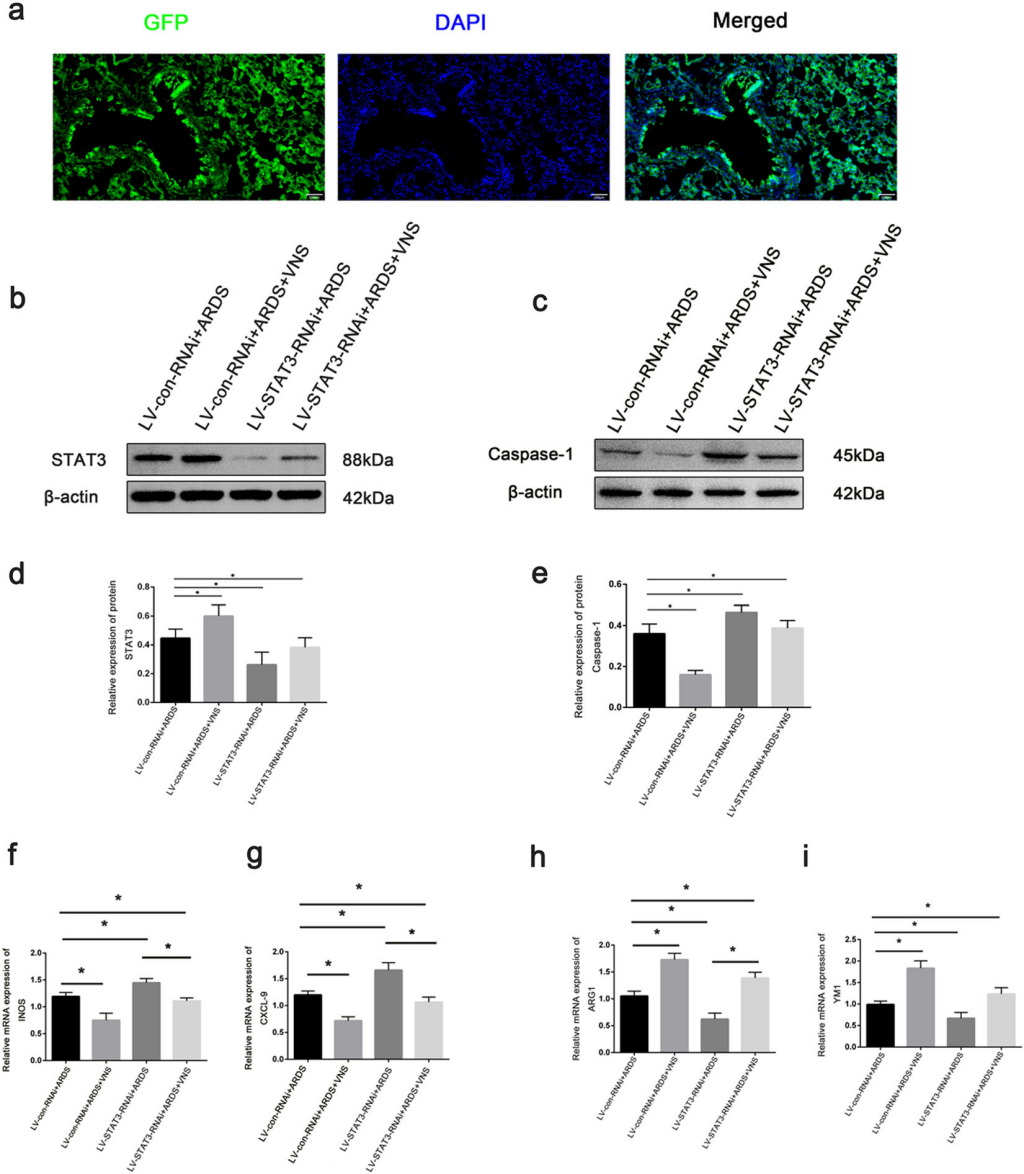
Knockdown of STAT3 attenuates VNS’s effects on STAT3, Caspase-1, and M1M2 macrophage phenotypes. **a** Fluorescence images of GFP (green) in injected lung 7 days after lentiviruses were stereotaxically injected (scale bar = 100 μm). **b** Representative western blotting images of STAT3 expression in the LV-con-RNAi+ARDS group, LV-con-RNAi+ARDS+VNS group, LV-STAT3-RNAi+ARDS group, and LV-STAT3-RNAi+ARDS+VNS group (*n* = 6 biological replicates). **c** Representative western blotting analysis images of Caspase-1 expression in the LV-con-RNAi+ARDS group, LV-con-RNAi+ARDS+VNS group, LV- STAT3-RNAi+ARDS group, and LV-STAT3-RNAi+ARDS+VNS groups (*n* = 6 biological replicates). **d** Comparison of mean intensity ratios for STAT3. **e** Comparison of mean intensity ratios for Caspase-1. **f**–**i** The mRNA levels of the M1 macrophage markers, iNOS and CXCL-9, and those of the M2 macrophage markers, Arg1 and Ym1, were measured in the LV-con-RNAi+ARDS group, LV-con-RNAi+ARDS+VNS group, LV-STAT3-RNAi+ARDS group, and LV-STAT3-RNAi+ARDS+VNS group by qPCR and normalized to GAPDH mRNA levels (*n* = 6 biological replicates). The results are expressed as the mean ± SEM (*n* = 6 biological replicates); *p < 0.05, **p < 0.01.

Immunofluorescence results showed successful transfection of lentivirus in the lung tissue (Fig. 5a). Compared with LV-con-RNAi+ARDS rat, the expression of STAT3 was successfully reduced after STAT3 knockdown (Fig. 5b, d). Compared with VNS treatment alone, the level of IL-1β and TNF-α increased, while that of IL-10 decreased in the ARDS model rats with low STAT3 expression (Fig. 1b–d), and the expression of Caspase-1 was increased in the lung tissue (Fig. 5c, e). M1 macrophages were observed to increase, while the proportion of M2 macrophages was decreased (Fig. 5f–i). These results suggest that the STAT3 is involved in regulating the inflammatory response in ARDS. Moreover, VNS increases STAT3 expression, affects the phenotype transformation of macrophages and the pyroptosis of lung tissue cells, and regulates the expression of pro-inflammatory factors and anti-inflammatory factors, which form an important mechanism for reducing the inflammatory response in ARDS.

## Discussion

During the inflammatory reaction in ARDS, acetylcholine increases to activate the α7nAchR present on the membrane of inflammatory cells (including macrophages and neutrophils) as well as STAT3. Recent studies have shown that α7nAchR can downregulate the activation of inflammasomes^26–29^. It inhibits the secretion of pro-inflammatory cytokines and chemokines in inflammatory cells including alveolar macrophages and reduces lung inflammation and injury. Using the LPS-induced ARDS rat model, we found that the administration of an α7nAchR agonist in ARDS rats can reduce lung injury, while vagus nerve cutting and the administration of an α7nAchR inhibitor can aggravate the pulmonary inflammatory response, suggesting the presence of a pulmonary parasympathetic inflammatory reflex in the inflammatory response associated with ARDS. VNS can activate and enhance the activity of CAIP to reduce inflammation^30,31^. Our results suggest that VNS activates the α7nAchR receptor, affects macrophage transformation and lung tissue cell pyroptosis by activating STAT3, reduces the release of pro-inflammatory cytokines like TNF-α and IL-1β, and increases the release of anti-inflammatory cytokines like IL-10, all of which are important mechanisms for reducing the inflammatory response.

In the initial inflammatory response phase of ARDS, macrophages play an important role in promoting tissue repair and reducing the inflammatory response^32,33^. The diversity of macrophage functions is attributed to the plasticity of macrophage phenotypes, including classically activated M1 macrophages and alternatively activated M2 macrophages^19,33,21^. Macrophages are involved in both the development and resolution of ARDS, in that their function in disease is determined by their polarization state^33^, M1 macrophages can polarize Th1 cells and secrete TNF-α to promote inflammation, while M2 macrophages can induce Th2 cells to secrete IL-10 to inhibit inflammation and promote inflammatory damage repair^33^. After subjecting ARDS model rats to VNS, it was found that M1 macrophages transformed into M2 macrophages. M1 macrophages that play a pro-inflammatory role are mostly found in the inflammatory stage of ARDS, while M2 macrophages that play an anti-inflammatory role in the lung are increased after VNS. The phenotypic changes of macrophages M1 and M2 were also affected after the intervention of GTS-21 and MLA and the reduction of STAT3 expression with lentivirus. These results suggest that CAIP and STAT3 are involved in the regulation of macrophage transformation from the M1 to M2 phenotype. M1/M2 polarization in our results was determined indirectly by gene expression, and that the confirmation of the data on macrophage polarization in the future by flow cytometry would strengthen the findings.

The α7nAchR agonists and inhibitors have been shown to directly affect the secretion of pulmonary inflammatory factors and regulate STAT3 located downstream. STAT3 is a signal transduction and transcriptionally activated cytoplasmic protein that can be induced by a variety of cytokines or growth factors^34–36^. The activated STAT3 protein enters the nucleus and binds to the regulatory region of the target gene, and regulates the expression of specific nucleotide sequences in promoter regions of genes including TNF-α, iNOS, IL- 6, etc.^37^. It participates in biological processes, such as the immune regulation of cells^37^. After the expression of STAT3 was reduced by lentivirus knockdown, the effect of CAIP was effectively inhibited, this suggests that VNS may enhance CAIP activity to antagonize inflammation by activating STAT3.

Pyroptosis is a newly discovered programmed cell death pathway that plays an important role in the inflammatory response of ARDS^22,38^. The inflammatory factor, IL-1β, and the pathway Caspase-1 associated with pyroptosis have been shown to be involved in the inflammatory response of ARDS^39,40^. We found that VNS can reduce the expression of Caspase-1 and IL-1β in ARDS model rats; the expression of IL-1β and Caspase-1 can be interfered by α7nAchR agonists and inhibitors as well as by cutting off the vagus nerve. These findings suggest that VNS can influence pyroptosis of inflammatory cells through CAIP and that this process may be important for its anti-inflammatory effect; the potential mechanism underlying this process thus needs further study.

In summary, VNS plays an anti-inflammatory and reparative role in the ARDS rat model. VNS activates α7nAchR on the inflammatory cell membrane, affects STAT3 expression, regulates inflammatory cell pyrogen and M1 macrophage to M2 macrophage transformation, and influences the secretion of pro-inflammatory factors and anti-inflammatory factors; these processes may constitute the main mechanism of VNS action in ARDS. Our findings provide theoretical support for the future regulation of CAIP activity by VNS in the treatment of ARDS. It has been reported recently that activating the cholinergic anti-inflammatory path might be considered to alleviate severe COVID-19 with or without concurrent oxygen-induced lung injury^41^, and GTS-21 may provide a novel approach for developing therapies to treat oxidative stress-induced inflammatory lung injury in patients on oxygen therapy^42^. Our experiment has proved that the combination of GTS-21 and VNS can better reduce the inflammatory response in ARDS rats, which may provide a new idea for the treatment of COVID-19.

## Materials and methods

### LPS-induced ARDS model

Healthy adult male Sprague–Dawley rats (200–250 g) were purchased from the Experimental Animal Centre of Chongqing Medical University (Chongqing, China). All rats were housed at a 12 h/12 h light/dark cycle room maintained at 21–22 °C with a relative humidity of 60% and allowed free access to food and water. All animal experimental procedures were performed in accordance with the Guide for the Care and Use of Laboratory Animals by the National Institutes of Health, and were approved by the Ethics Committee of Chongqing Medical University. The ARDS model was established as described previously^43^. Briefly, the rats were anesthetized with 3% sodium pentobarbital and LPS (*Escherichia coli* LPS serotype 0111: B4 dissolved in phosphate-buffered saline (PBS)) was administered via intratracheal instillation at the dose of 2 mg/kg. The blood and lung samples were collected at 8 h after LPS administration and VNS treatment. The rats were randomized, and the researcher who conducted the experiments was blinded to the treatments.

### Electrical stimulation of the vagus nerve

The rats were deeply anesthetized and the left cervical nerve was prepared free from the carotid artery followed by ligation with silk suture. In the VNS animals, the nerve was stimulated for 10 min at 5 V, 5 Hz, 2 ms^32,33^ after the establishment of ARDS rat model; in vagotomized animals, both the left and right vagus nerves were transected (bilateral vagotomy). For sham stimulation of the vagus nerve, the cervical skin of the control rats was opened and covered by moist gauze. During the experiment, the body temperature of the animals was maintained at approximately 37 °C using an electrothermal pad. All animals were sacrificed by exsanguination after 2 h and the blood and lung tissues were collected.

### Administration of α7nAchR agonist and antagonist

For the α7nAchR intervention study, the corresponding antagonist MLA (6 mg/kg, MCE, USA)^44,45^, and agonist GTS-21 (4 mg/kg, MCE, USA)^42,46,47^ were used. According to a previously described method in a study, we diluted MLA and GTS-21 in PBS and injected the rats intraperitoneally 30 min before administering LPS.

### Lentivirus administration

To knock down the expression of STAT3, a small interfering RNA (siRNA) with the following sequence was used: TGGAGGAGAGGATCGTGGATCTGTT. The pHBLV-U6-MCS-CMV-ZsGreen-PGK-PURO lentiviral vector was used in the study. Lentiviral vectors expressing STAT3-RNAi (LV-STAT3-RNAi) were supplied by Hanbio Tech Co., Ltd. (Shanghai, China). Lentiviral vectors coding for GFP were used as the control (LV-con-RNAi). Seven days before ARDS, lentiviruses were injected intratracheally into the lung tissue.

### H&E staining

Left lung lobes were isolated, fixed in 3.7% paraformaldehyde, embedded in paraffin wax, cut into 5-μm sections, and stained with H&E.

### Enzyme-linked immunosorbent assay

After the animals were exsanguinated, the BALF was collected by washing the lungs with 0.5 ml of sterile saline and withdrawing the fluid (three times). The BALF was used to assay the levels of TNF-α, IL-1β, and IL-10 using the respective commercially available ELISA kits (R&D, Minneapolis, MN, USA) according to the manufacturer’s instructions.

### Western blotting

Proteins were extracted from the lung tissues of animals belonging to different groups using a whole protein extraction kit (Beyotime, China). The protein concentration was measured using the BCA protein assay reagent (P0010S, Beyotime, China). After denaturation by boiling, all protein samples were stored at −80 °C until further analysis. Equal amount of protein was loaded into each well for separation via SDS-PAGE, electrophoresed on 10% or 12% separating gels, and transferred onto PVDF membranes (IPVH00010, Millipore, USA). The membranes were blocked with 5% skim milk and incubated overnight at 4 °C with the following primary antibodies: anti-STAT3 (1:1000, ab119352, abcam), anti-Caspase-1 (1:1000, BS90183, Bioworld Technology, Inc. USA), and anti-β-actin (1:1000, GB12001, Servicebio, China). β-actin was used as a loading control. After washing with TBST, the membranes were incubated with horseradish peroxidase conjugated goat anti-rabbit IgG antibody (1:4000, SA00001-2, Proteintech, China) or anti-mouse IgG antibody (1:4000, SA00001-1, Proteintech, China) for 1 h at room temperature. Then, the membranes were washed with TBST and visualized using an enhanced chemiluminescence reagent (P0018FS, Beyotime, China). Images were captured by a Fusion FX5 analysis system (Vilber Lourmat, F-77601 Marne-la-Vallée cedex 3, France) and quantified using Quantity One software (Bio-Rad Laboratories, USA).

### Quantitative real-time PCR

Total RNA was isolated from the lung tissues using TRIzol reagent (Invitrogen, Carlsbad, CA, USA) according to the manufacturer’s instructions and quantified using a Nanodrop 2000 spectrophotometer (Thermo Scientific). Subsequently, 1 μg of total RNA was used as a template for amplifying the cDNA using a HiScript 1st Strand cDNA Synthesis Kit (Vazyme Biotech, Nanjing, China). INOS, CXCL-9, YM1, and ARG1 gene expression was detected using a HiScript II One Step quantitative real-time PCR SYBR Green Kit (Vazyme Biotech) and a StepOne Real-Time PCR System (Applied Biosystems, Foster City, CA, USA). The relative gene expression was normalized to that of the endogenous control GAPDH using the comparative Ct (ΔΔCt) method. The primer sequences were designed as follows: INOS, 5ʹ-TCGGGCTGAAGTGGTATGC-3ʹ (forward primer) and 5ʹ-CAGAAGTCTCGGACTCCAATCT-3ʹ (reverse primer); CXCL-9, 5ʹ-GCCAAGGCACATTCCACTACA-3ʹ (forward primer) and 5ʹ-GGCAGGTTTGATCTCCGTTC-3ʹ (reverse primer); ARG-1, 5ʹ-CAAGACAGGGCTACTTTCAGGAC-3ʹ (forward primer) and 5ʹ-GATTACCTTCCCGTTTCGTTCC-3ʹ (reverse primer); YM1, 5ʹ-TGGAGGCTGGAAGTTTGGAT-3ʹ (forward primer) and 5ʹ-TGCACCAGGACACTGAAGAGA-3ʹ (reverse primer).

### Immunofluorescence

Lung tissues were fixed with 3.7% paraformaldehyde, and permeabilized with 0.5% Triton X‑100. After washing with PBS, sections were blocked in normal goat serum for 1 h. Then, the sections were incubated overnight at 4 °C with the anti-p-STAT3 primary antibody (1:50). The next day, the sections were washed with PBS and incubated with a mixture of goat anti-rabbit IgG-CFL 488 (1:100, sc-362262, Santa Cruz Biotechnology, USA) and goat anti-mouse IgG-CFL 555 (1:200, sc-362267, Santa Cruz Biotechnology, USA) at 37 °C for 1 h in the dark. Then, the sections were counterstained with DAPI for observing the nuclei. Images were captured by a confocal laser scanning microscope (A1 + R, Nikon, Tokyo, Japan).

### Statistical analysis

The data are expressed as the mean ± SD. GraphPad Prism 6.0 was used for graphing, and SPSS 25.0 was used for statistical analyses. One-way or two-way analysis of variance followed by the Tukey’s post-hoc multiple comparison test was used to analyze the differences between groups. Differences in values were considered significant at *p* < 0.05.
